# Deep neural networks for inverse design of multimode integrated gratings with simultaneous amplitude and phase control

**DOI:** 10.1515/nanoph-2024-0667

**Published:** 2025-03-14

**Authors:** Ali Mohajer Hejazi, Vincent Ginis

**Affiliations:** Applied Physics, Vrije Universiteit Brussel, 1050 Brussel, Belgium; Data Analytics Lab/Applied Physics, Vrije Universiteit Brussel, Pleinlaan 2, 1050 Brussel, Belgium; Harvard John A. Paulson School of Engineering and Applied Sciences, Harvard University, 29 Oxford Street, Cambridge, MA 02138, USA

**Keywords:** artificial neural networks, inverse design, grating, mode converter

## Abstract

We present a photonic mode converter based on a grating structure, modeled and inversely designed by deep neural networks. The neural network maps the physical parameters of the grating to the grating responses, i.e., complex scattering parameters representing the reflected modes from the grating structure. We design different neural networks to output the magnitudes and the phases of the scattering parameters associated with the multiple reflected modes. Following the training process, we use the trained networks to perform inverse design of the grating based on the desired magnitudes of the scattering parameters. The inverse design effort provides a full control on the magnitudes and the phases of the reflected modes from the mode converter. Our techniques help in creating a rich landscape of multiple interfering waves that provide higher control on optical near fields, complex resonators, and their relevant nanophotonic applications.

## 1 Introduction

Light–matter interactions in photonic devices bring about the emergence of several interdependent physical phenomena. The design of those devices relies on analytical knowledge, physical intuitions, and experiments.

In most design cases, practitioners resort to numerical simulations of the electromagnetic problems arising from light–matter interactions in photonic devices. Since last two decades, in addition to discovering and creating new functionalities in photonic systems, there have been vigorous efforts to maximize the capabilities of the devices through various optimization and inverse design techniques. Those efforts have been inevitable since novel photonic technologies often require a combination of small-size integration, sub-wavelength features, efficient utilization of optical nonlinearity, broadband capabilities, etc. [1], [2], [3], [4], [5], [6].

Inverse problems, in general, can be understood from the perspective of forward problems. Forward problems, particularly in photonics and electromagnetism, involve analytically and numerically solving the Maxwell’s equations to obtain the responses of light interactions with physical domains such as waveguides, couplers, and nanostructures. These responses might be in the forms of scattering coefficients, radiation patterns, bandwidth, etc. On the other hand, in inverse problems, one or more desired responses are selected, then an optimization algorithm tries to achieve the desired response through systematic fine-tuning of the material and geometrical parameters of the physical domain [5]. Inverse problems often involve the maximization or minimization of one or multiple objective functions subject to some constraints. There are several techniques to achieve inverse optimal design of photonics devices. Among those techniques, we can mention evolutionary algorithms such as genetic algorithm [7], [8], [9], [10], [11] and particle swarm optimization [12], [13], [14], [15], [16], gradient-based methods for topology optimization [17], [18], [19], [20], [21], [22], optimization via deep neural networks [23], [24], [25], [26], etc.

Due to the power of deep learning techniques in tackling both forward and inverse problems, they have vigorously expanded since the last decade. Furthermore, another reason for the expansion is the emergence of computational hardware, like graphical processing units [27], [28], and software technologies [29] that have facilitated deep learning methods.

The deep neural networks, in forward problems, could be exploited to map a collection of feature vectors of variables – input independent variables – to the output vectors that are paired with the input vectors. A trained neural network could predict new outputs from new input provided that the input is within the space of the training data set. The capability of neural networks in creating maps from the input features to the output labels could be boosted by using a large number of data samples in the training process. The training mechanism in the neural network for a forward problem is initiated by feeding data at the network’s input. Initially, the parameters of the network (weights and biases) are randomly generated. The network’s output is estimated based on the generated parameters and the input data features. The estimated values of output would be compared with the actual labels from the dataset via a chosen loss function. The gradients of the loss function with respect to the weights and the biases are calculated, and the parameters of the network are adjusted according to the procedure stated in the gradient descent algorithm. This procedure would repeat until the value of the loss function becomes less than a defined threshold number.

On the other hand, an inverse design problem might be solved with the assistance of a trained deep neural network in which the weights and biases are fixed values. First, the desired values for the dependent variables are selected. Then, the mechanism is initiated by generating a random set of values as input features of the neural network. The trained neural network estimates the corresponding outputs. In this inverse design problem, gradient descent is also the core algorithm to achieve optimal results. A loss function is defined, and the error value between the desired output and the calculated output is obtained from the loss function. The derivatives of the loss function with respect to the input features are computed. The updated values of the input features can be obtained according to the algorithm. This procedure repeats until the value of the loss function becomes smaller than a defined threshold [5]. Two lucid examples of using the mentioned mechanism in nanophotonic inverse design are the studies conducted by Purifoy et al. [30] and Lenaerts et al. [31], where the former used deep neural networks for forward approximation of scattered fields from a multi-layered dielectric particle. Furthermore, they used the trained neural network in inversely design of the particle for desired scattered responses. In the latter research work, a deep neural network was utilized first for modeling the transmission spectra of a Fabry–Perot resonator. The trained model was exploited to optimize the physical parameters satisfying the desired transmission spectra.

This article uses inverse design for grating mode converters in order to precisely engineer the amplitudes and phases of multiple reflected modes at once. This is crucial in advanced applications such as designing the intricate near fields resulting from the interference of cascaded counterpropagating modes created by a succession of engineered mode converters [32], and creating novel optical resonators with unconventional electromagnetic properties [33].

All the modeling through deep neural networks and the inverse design process are performed by taking advantage of the codes provided in well-established Python library Tensorflow [34]. We use Python libraries Pandas [35] and Scikit-learn [36] for data manipulation and some statistical calculations.

## 2 Deep neural network modeling of the grating waveguide mode converter

Photonic waveguide gratings have been exploited in numerous applications such as optical couplers [37], [38], wavelength filtering [39], Bragg-reflection devices [40], mode-converting devices [41], field enhancement and nonlinear optical phenomena via resonant effects in grating dielectric waveguide [42], [43] etc. Cascaded mode-conversion [32], [33] is another intriguing application of waveguide gratings which is also the subject of this letter.

The mode-conversion capability of a waveguide grating emanates from the violation of mode orthogonality between guided modes due to the presence of the longitudinal periodic perturbation. The longitudinal phase matching condition required to be satisfied for the mode conversion is given by [41], [44]:

(1) $$\beta_{\mu }+\beta_{\nu }-m\left(\frac{2\pi }{\Lambda }\right)=0,$$

where *β*_*μ*_ and *β*_*ν*_ are the propagation constants of the excited mode and the converted mode respectively. The parameters Λ and *m* are the grating period and an arbitrary integer, respectively.

The dielectric waveguide is designed to guide the first three TE modes at wavelength 1,550 nm. Silicon, with refractive index equal to 3.48, has been selected as the dielectric material to be utilized for the waveguide and the grating. The dielectric portion of the waveguide is periodically removed to create the grating structure. The area surrounding the structure is determined to be vacuum. The whole structure is illustrated in Figure 2n on the bottom. The symbols Λ, *d* and *t* are respectively denoting the grating period, the depth of corrugations and the waveguide thickness.

**Figure 1:**
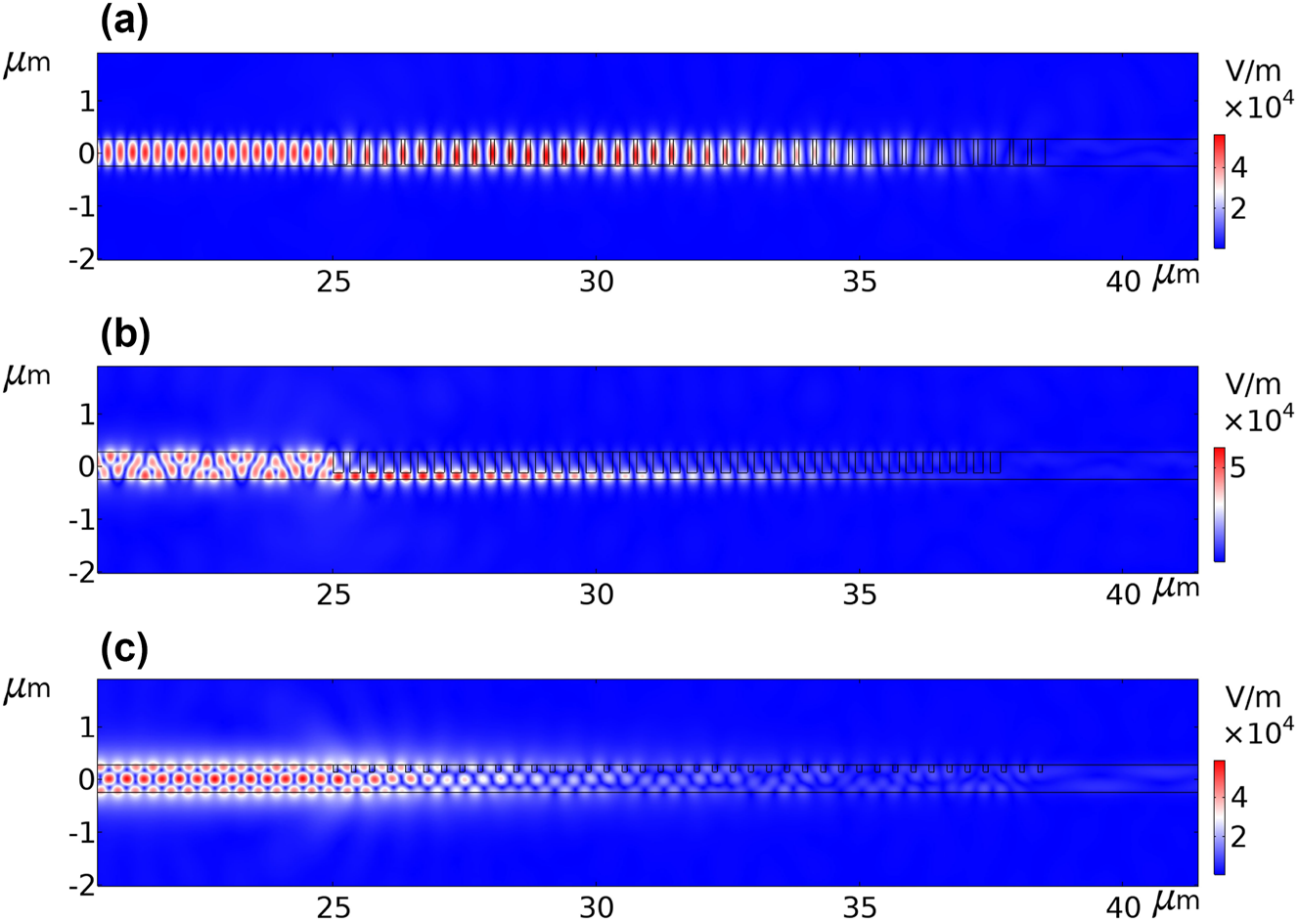
The plots illustrate the electric field norms in the presence of the grating mode-converter. The grating structure is distributed in the horizontal direction from *x* = 25 μm to around *x* = 38.5 μm. The first waveguide mode is excited from the left side in all plots. In all three scenarios, the figures illustrate a substantial decrease in the electric field on the right side of the gratings. This observation indicates that a substantial fraction of the energy in the field is reflected by the grating mode-converter. (a) Demonstrates the reflection of the 1st mode. The magnitude of *S*_11_ is equal to 99 %. The geometrical properties that result in the value of |*S*_11_| are as follows: the period **Λ** is 340 nm, the corrugation depth **d** is 490 nm, and the duty cycle is 0.216. (b) The plot illustrates the conversion of the 1st mode to the 2nd mode when the 1st mode is excited. The converted mode is reflected back from the grating. The magnitude of *S*_21_ quantifies the extent to which the incident field is converted into the 2nd mode field. In this case, the magnitude of *S*_21_ is equal to 86.5 %. The grating’s geometric properties that contribute to this outcome are as follows: the period (**Λ**) is 320 nm, the corrugation depth (**d**) is 390 nm, and the duty cycle is 0.417. (c) This plot shows the conversion of the 1st mode to the 3rd mode when the 1st mode is excited. The |*S*_31_| indicates the how much of the incident mode field is converted to the 3rd mode. In this case the parameter |*S*_31_| is equal to 97.5 %, and the relevant geometrical properties of the grating are as follows: the period **Λ** is 343 nm, the corrugation depth **d** is 130 nm, and the duty cycle is 0.74.

**Figure 2:**
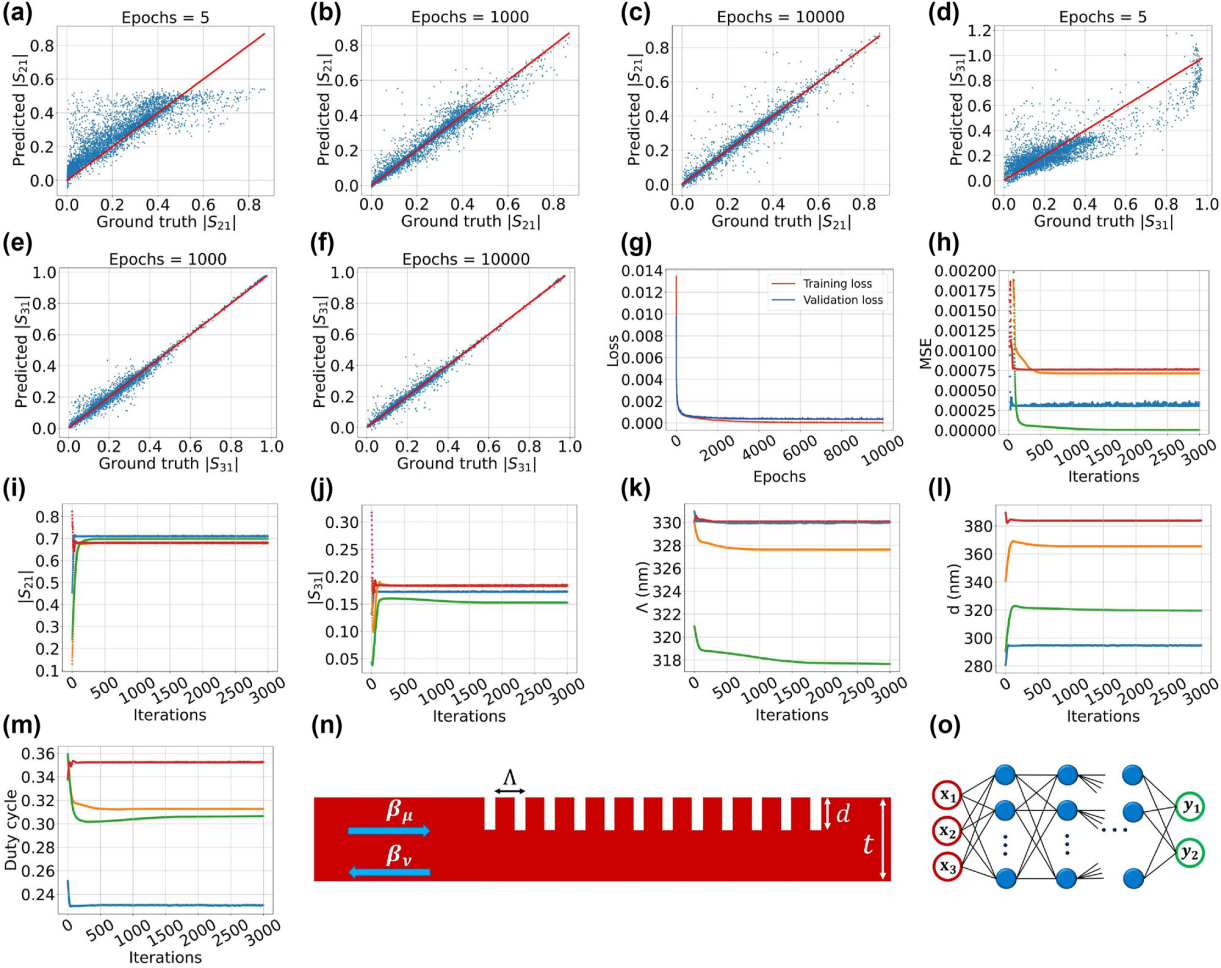
The plots are in three groups. The first group (a–g) shows the performance of a double-output neural network created to output the two scattering parameters’ magnitudes. The second group (h–m) demonstrates the changes in various parameters during the inverse design process using the double-output neural network. The third group (n–o) shows the appearance of the grating mode converter and the utilized neural network. (a)–(c) Illustration of the scatter plots of the predicted |*S*_21_| over their actual values for different training epochs of 5, 1,000, and 10,000. Similar plots for |*S*_31_| are also depicted in (d)–(f), respectively. (g) Shows plot of the training (red) and validation (blue) losses over the 10,000 epochs. (h)–(m) Depict the results obtained from the inverse design of the grating mode converter via the trained neural network for desired magnitude of the scattering parameters |*S*_21_| = 0.7 and |*S*_31_| = 0.15. The inverse design algorithm is used many times leading to four different fairly accurate solutions. Each figure shows the evolutions of different variables such as value of the mean squared-error function, the scattering parameters and the physical features of the grating during optimization process. It is noteworthy that each colored curve in the figures is assigned to the same inverse design solution. (n) Illustration of the shape of the grating mode-converter connected to a waveguide. The waveguide thickness is equal to 520 nm. For the simulations to establish the data set, the ranges of the grating period, the corrugation depth, and the duty cycle are respectively chosen as 315–350 nm, 10–520 nm, and 0.1–0.9. (o) The neural network architecture used for the forward and the inverse problems is illustrated. It possesses 3 input nodes – period “Λ”, corrugation depth “d”, and duty cycle – and 2 output nodes being magnitudes of the scattering parameters |*S*_21_| and |*S*_31_|.

The structure is simulated by exciting the first *TE* mode of the waveguide. The scattering coefficients *S*_11_, *S*_21_ and *S*_31_ are calculated. The *S*_11_, *S*_21_ and *S*_31_ are representing the reflection of the 1st mode, the reflection of the 2nd mode when the 1st mode is excited, and the reflection of the 3rd mode when the 1st mode is excited, respectively. In the simulation part, we sweep the parameters Λ, *d*, and duty cycle over a range of values and compute the scattering parameters. The duty cycle refers to the portion of the grating period that constitutes the grating material (Silicon). The caption of the Figure 2 provides more information about the specified ranges of the sweeping parameters. For the electromagnetic simulations of the grating mode converter, we employed COMSOL Multiphysics which is a finite element software. A data set comprising 50,545 samples is created from the simulations. Figure 1 illustrates the norms of the electric fields associated with three of these samples. Figure 1a–c depict the grating mode-converter for highly efficient conversion of the 1st mode to the 1st, the 2nd, and the 3rd mode, respectively.

The input of the deep neural network (DNN) consists of three nodes representing the physical features of the grating i.e. the period, corrugation depth, and the duty cycle. This DNN is a supervised learning model, so that the labels corresponding to the input features are selected as two of the scattering parameters (for instance |*S*_21_| and |*S*_31_|). The DNN architecture is illustrated in Figure 2o. The DNN possesses five hidden layers each with 600 nodes. The activation function designated for the first and the last two hidden layers is the ReLU function. The Sigmoid function is specified as the activation function of the hidden layer located at the middle of the DNN. In the training process, a batch size of 128 is used. The loss function for the forward DNN model is designated as the logarithm of hyperbolic cosine which is given by:

(2) $$\begin{array}{ll} L & =\frac{1}{2n}\overset{n}{\underset{i=1}{\sum }}\;\left(\log\;\left(\frac{e^{\left(y_{P_{1}}^{i}-y_{G_{1}}^{i}\right)}+e^{-\left(y_{P_{1}}^{i}-y_{G_{1}}^{i}\right)}}{2}\right)\right.\\ & \;+\left.\log\;\left(\frac{e^{\left(y_{P_{2}}^{i}-y_{G_{2}}^{i}\right)}+e^{-\left(y_{P_{2}}^{i}-y_{G_{2}}^{i}\right)}}{2}\right)\right), \end{array}$$

where *n* denotes the number of samples from the training set. The symbols $y_{P_{1}}$ and $y_{P_{2}}$ indicate the DNN output predictions. The corresponding ground truth values are represented by $y_{G_{1}}$ and $y_{G_{2}}$ . The factor 2 in loss function relation 2 denotes the numbers of the output nodes of the DNN. The size of the training set is determined as 80 % of the number of samples in the data set.

The scatter plots of the predicted |*S*_21_| and |*S*_31_| over their actual values for various training epochs are depicted in Figure 2a–f. The Figures show how the DNN’s prediction performance improves with increasing epoch number. The points in the scatter plots are from a test set that include 10 % of the whole data set. Figure 2g shows the decreasing trend of the loss function with increasing epochs. The red curve depicts the loss-function values for the training set, which constitutes 80 % of the whole data set. The blue curve demonstrates the loss function values associated with the validation dataset. Both curves suggest low values at their end points without significant divergence between them.

We calculate some statistical measures such as “Mean-squared error” (MSE), “*R* ^2^ Score”, and “Explained variance score” (EVS) in order to quantitatively assess the DNN performance in prediction of |*S*_21_| and |*S*_31_|. These statistical measures are listed in Table 1. The values in the Table are attributed to models depicted in Figure 2c and f. The *R* ^2^ score is a metric that measures how well the variability of the independent variables (DNN inputs) can account for the variability of the dependent variables (DNN outputs). In an ideal case, *R* ^2^ score is equal to unity that implies the trained model has zero error in its prediction ability. Another statistical measure mentioned in Table 1 is EVS indicating how much the prediction errors are dispersed in a regression model. EVS ranges from 0 to 1, with unity being the ideal number. A large value of EVS means that the variance of the prediction error is small compared to the variance of the actual labels which is desired.

**Table 1:** The list of the statistical measures for the assessment of the double-output DNN model for the prediction of |*S*_21_| and |*S*_31_|. MAE, MSE, and EVS stand for mean-absolute error, mean-squared error, and explained-variance score respectively.

	MAE	MSE	*R* ^2^ score	EVS
For |*S*_21_|	0.014353	0.001106	0.960086	0.960177
For |*S*_31_|	0.008272	0.000293	0.991627	0.991627

## 3 Inverse design assisted by the trained neural network model

The trained DNN with fixed weights and biases could be exploited for the inverse design of the grating mode converter. We define the desired values of the two scattering parameters, for instance |*S*_21_| and |*S*_31_|. The initial step in the inverse design algorithm is to randomly generate the physical features at the input nodes of the trained DNN. The trained DNN estimates the scattering parameters based on the features. The estimated scattering parameters are evaluated by comparing them with the desired scattering parameters using a loss function such as mean squared-error (MSE), that has the given mathematical form:

(3) $$L=\frac{1}{2}\left({\left(\left|S_{21}^{m}|-|{\hat{S}}_{21}\right|\right)}^{2}+{\left(\left|S_{31}^{m}|-|{\hat{S}}_{31}\right|\right)}^{2}\right),$$

where the terms with the hat are associated with the constant desired parameters, and the ones with “*m*” superscript are the DNN outputs which are the functions of the three physical features of the mode converter. Based on the procedure in the gradient descent algorithms, it is required to compute the derivatives of the loss function with respect to the DNN’s input features. Subsequently, the updated version of the features is computed based on the old values of the features and the values obtained from the derivatives. For the step size (learning rate) in the gradient descent, we use the Adam optimizer [45] with learning rate parameter equal to 0.001. This process continues until the value of the loss function decreases below a specified threshold.

Let the selected loss function and the output function of the trained neural network be represented by the symbols **L** and **J**, respectively. The mathematical formulation for the update equations to update the values of the grating geometrical characteristics is as follows:

(4) $$L=\frac{1}{n}\sum {\left(J\left(\Lambda_{m},d_{m},t_{m}\right)-J_{\text{desired}}\right)}^{2},$$

(5) $$\Lambda_{m+1}=\Lambda_{m}-\alpha \frac{\partial \;L}{\partial \;\Lambda_{m}},$$

(6) $$d_{m+1}=d_{m}-\alpha \frac{\partial \;L}{\partial \;d_{m}},$$

(7) $$t_{m+1}=t_{m}-\alpha \frac{\partial \;L}{\partial \;t_{m}},$$

where the symbol *m* denotes the iteration number in the gradient descent process. The **J**_desired_ is the target value for which we aim to achieve through inverse design. The trained DNN, represented by the symbol *J*, is a function of its inputs: grating period (Λ), corrugation depth (*d*), and duty cycle (*t*).

The results of the inverse design for simultaneous desired magnitudes of the scattering parameters |*S*_21_| = 0.7 and |*S*_31_| = 0.15 are illustrated in Figure 2h–m. These curves are the results of running the optimization algorithm several times in which we obtain eight different satisfactory results that approximately meet the requirements. The Figure 2h–m shows four of these eight cases to keep the graph neat. We choose 3,000 as the iteration counts in the optimization process. Each time utilization of the optimization algorithm takes around 1 min. The changes in the MSE loss function, |*S*_21_| and |*S*_31_| are depicted in Figure 2h–j. Moreover, the gradual developments of the period, corrugation depth, and duty cycle from their initial random guess to their final optimal values are illustrated in Figure 2k–m. Table 2 lists the final optimal values of the grating physical features from the eight optimal cases. The table also includes four cases depicted in Figure 2, which are written in non-black colors. These features are also tested in COMSOL software and the results are listed in the Table 2. The computed scattering parameters in the software (|*S*_21*c*_|, |*S*_31*c*_|) are listed beside the calculated scattering parameters obtained from the DNN model (|*S*_21*m*_|, |*S*_31*m*_|).

**Table 2:** This table lists the values of the grating physical features obtained from the inverse design with desired scattering parameters |*S*_21_| = 0.7, |*S*_31_| = 0.15. We use the COMSOL software to simulate the grating with the obtained physical features. The computed scattering parameters through the software, indicated by |*S*_21*c*_|, |*S*_31*c*_|, are listed beside the estimated scattering parameters, denoted by |*S*_21*m*_|, |*S*_31*m*_|, obtained from the double-output DNN model. The colored rows are related to the inverse design curves in Figure 2h–m. DC stands for duty cycle.

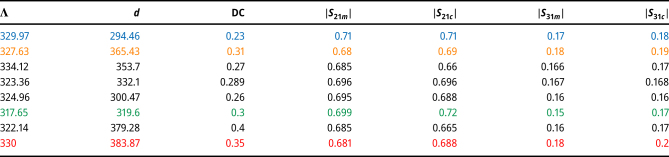

## 4 Forward modeling and inverse design via triple-outputs DNN

We also conduct inverse design of the grating mode-converter to achieve desired values of |*S*_11_|, |*S*_21_|, and |*S*_31_| simultaneously. The first step is to train a new DNN model that consists of three output nodes. This model will be used to estimate the magnitude of the scattering parameters. The DNN also has five hidden layers, each consisting of 600 nodes, and utilizes the same activation functions ReLU and Sigmoid.

The key distinction between the double-output and the triple-output DNN, aside from the number of output nodes, is that the latter exploits a dropout layer after each hidden layer. The dropout layer helps prevent the DNN model from overfitting by randomly deactivating some nodes in each layer in both forward and backward propagation. In this case, the droupout layer randomly deactivate the 5 % of the nodes in each layer.

The loss function utilized in the training process of triple-output DNN is defined as follows:

(8) $$\begin{array}{cc} L & =\frac{1}{3n}\overset{n}{\underset{i=1}{\sum }}\;\left(\log\;\left(\frac{e^{\left(y_{P_{1}}^{i}-y_{G_{1}}^{i}\right)}+e^{-\left(y_{P_{1}}^{i}-y_{G_{1}}^{i}\right)}}{2}\right)\right.\\ & \;+\log\;\left(\frac{e^{\left(y_{P_{2}}^{i}-y_{G_{2}}^{i}\right)}+e^{-\left(y_{P_{2}}^{i}-y_{G_{2}}^{i}\right)}}{2}\right)\\ & \;+\log\left.\;\left(\frac{e^{\left(y_{P_{3}}^{i}-y_{G_{3}}^{i}\right)}+e^{-\left(y_{P_{3}}^{i}-y_{G_{3}}^{i}\right)}}{2}\right)\right), \end{array}$$

where the factor 3 in the denominator represents the number of nodes in the output layer of the DNN.

**Figure 3:**
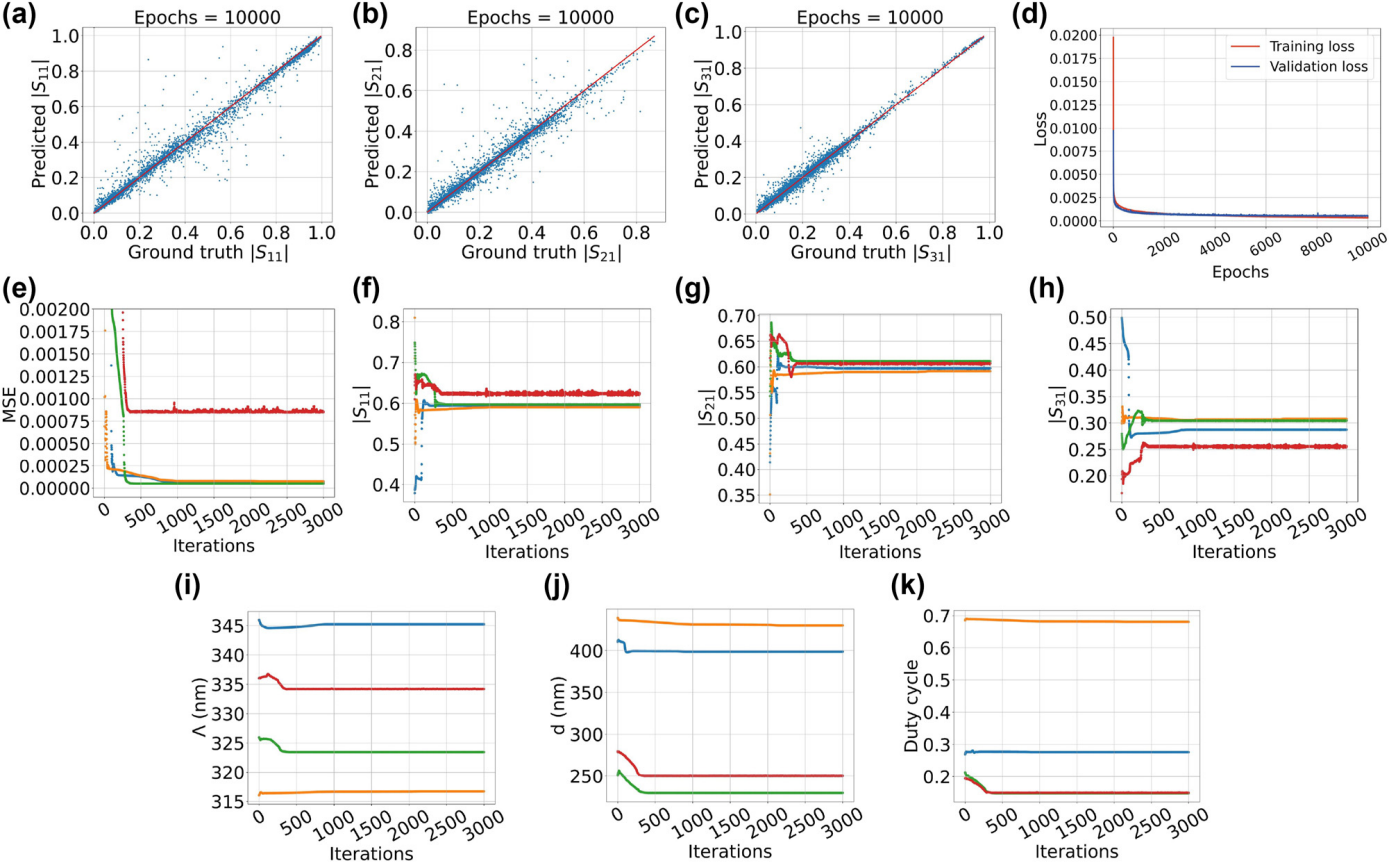
There are two groups of plots. The first group (a–d) demonstrates the performance of a triple-output neural network designed to produce the magnitudes of three scattering parameters. The second group (e–k) illustrates the inverse design process employing the triple-output network, which shows the gradual variations of various parameters during the process. (a)–(c) Demonstrate the scatter plots of the predicted scattering parameters |*S*_11_|, |*S*_21_|, and |*S*_31_| over their actual values for the training epoch number equal to 10,000. The blue points represent the test set including 10 % of the data set. (d) Shows the plot of the training (red) and validation (blue) losses over the 10,000 epochs. (e)–(k) Demonstrate results obtained from the inverse design of the grating mode converter for desired scattering parameters |*S*_11_| = 0.6, |*S*_21_| = 0.6, and |*S*_31_| = 0.3. The inverse design algorithm is used several times leading to four different solutions. Each figure shows the changes of different variables such as MSE function, the scattering parameters and the physical features of the grating during optimization process. Each colored curve in the figures is associated with the same inverse design solution.

To evaluate the performance of the triple-output DNN, we calculate the aforementioned statistical measures which is listed in Table 3. The metrics EVS and *R* ^2^ score, for all three scattering parameters, are close to unity which is a sign of an accurate model. Figure 3a–c illustrate the scatter plots of the predicted |*S*_11_|, |*S*_21_| and |*S*_31_| over their ground truth values for the training epoch of 10,000. These scatter plots are helpful for qualitatively graphical evaluation of the DNN model. The training loss and the validation loss curves are also depicted in Figure 3d. The curves are almost overlapped and both are extremely close to zero at their final points.

**Table 3:** The list of the statistical measures for the assessment of the triple-output DNN model for the prediction of |*S*_11_|, |*S*_21_|, and |*S*_31_|. MAE, MSE, and EVS stand for mean-absolute error, mean-squared error, and explained-variance score, respectively.

	MAE	MSE	*R* ^2^ score	EVS
|*S*_11_|	0.018118	0.001730	0.984159	0.984177
|*S*_21_|	0.015257	0.001088	0.960734	0.960734
|*S*_31_|	0.012638	0.000419	0.988031	0.988114

Similar to the inverse design of the mode converter based on the double-output DNN, we could use the same procedure for the triple-output DNN. First, we need to specify the desired values of the scattering parameters |*S*_11_|, |*S*_21_|, and |*S*_31_|, where in this case they are selected to be 0.6, 0.6, and 0.3 respectively. We employ the optimization algorithm several times and obtain four cases that satisfy the constraints of the inverse design problem. The mathematical form of the inverse problem loss function is given by:

(9) $$\begin{array}{ll} L & =\frac{1}{3}\left({\left(\left|S_{11}^{m}|-|{\hat{S}}_{11}\right|\right)}^{2}+{\left(\left|S_{21}^{m}|-|{\hat{S}}_{21}\right|\right)}^{2}\right.+\left.{\left(\left|S_{31}^{m}|-|{\hat{S}}_{31}\right|\right)}^{2}\right) \end{array}$$

Figure 3e–k depict the evolution of the MSE loss, the three scattering parameters, and the grating physical features, respectively.

We use the achieved results of the inverse design problem in COMSOL software to check the accuracy of them. The results are listed in Table 4. It is clear that the model predictions and the results from the software are in agreement with one another.

**Table 4:** This table lists the values of the grating physical features obtained from the inverse design with desired scattering parameters |*S*_11_| = 0.6, |*S*_21_| = 0.6, |*S*_31_| = 0.3. The COMSOL software is exploited to simulate the grating mode converter with the obtained physical features. The computed scattering parameters through the COMSOL software are indicated by |*S*_11*c*_|, |*S*_21*c*_|, |*S*_31*c*_|. The columns denoted by |*S*_11*m*_|, |*S*_21*m*_|, |*S*_31*m*_| are the estimated results from the triple-output DNN model. The colored rows are associated with the inverse design solution curves in Figure 3e–k.

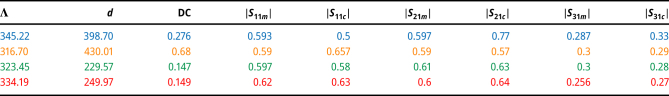

## 5 Modeling and inverse design of the phase of the scattering parameters

The preceding sections examine the process of modeling the magnitudes of the scattering parameters using deep neural networks that include multiple output nodes. Moreover, we perform the inverse design of the mode converter based on the trained neural networks. Each scattering parameter is a complex number. We also tried to model the phase of the scattering parameters through various neural network architectures. However, the efforts have not led to proper convergence.

For instance, Figure 5a depicts the training and validation losses over epoch numbers associated with a double-output DNN. The neural network is used to map the physical attributes of the mode converter with the amplitude and phase of *S*_21_. The ultimate values of the curves suggest that the losses are not sufficiently low. Furthermore, the curves diverge from each other that probably denote overfitting issue. Further evidence of the neural network’s limitations is shown in Figure 5b, where the blue dots represent the DNN’s predictions of the *S*_21_ phase compared to their actual values from a test set. Many of the blue points are distant from the red line, indicating the incapability of the DNN model. The statistical measures, including explained variance score (EVS), coefficient of determination (*R* ^2^ score), and mean squared error (MSE), for *S*_21_ phase in this example are equal to 0.679, 0.679, and 0.36, respectively. These values show the inadequacy of the DNN model. The reason behind this phase retrieval issue is partly the phase wrapping in the data set, partly the high sensitivity of the phase to the geometric features of the grating. Indeed, a sudden change in phase values causes a significant challenge for traditional neural networks to accurately model these values. In contrast to the phase, the amplitude is properly modeled by the double-output network, since the amplitude’s variation in the data set possesses smooth quality. These different behaviors of the phases and amplitudes result in highly nonlinear relations between the inputs and outputs of the neural network. In Figure 5c, the scatter plot shows the double-output DNN’s predictions of |*S*_21_| compared to their ground truth values for the test set. The EVS, *R* ^2^ score, and MSE values for |*S*_21_| are equal to 0.913, 0.913, and 0.0024, respectively.

We tried three other methods to tackle the phase retrieval. These include data augmentation, the use of alternative loss functions, and training a triple-output neural network mapping the geometrical features to the real part, imaginary part and the magnitude of the corresponding scattering parameter. We found that the latter method works better compared to the former two.

**Figure 4:**
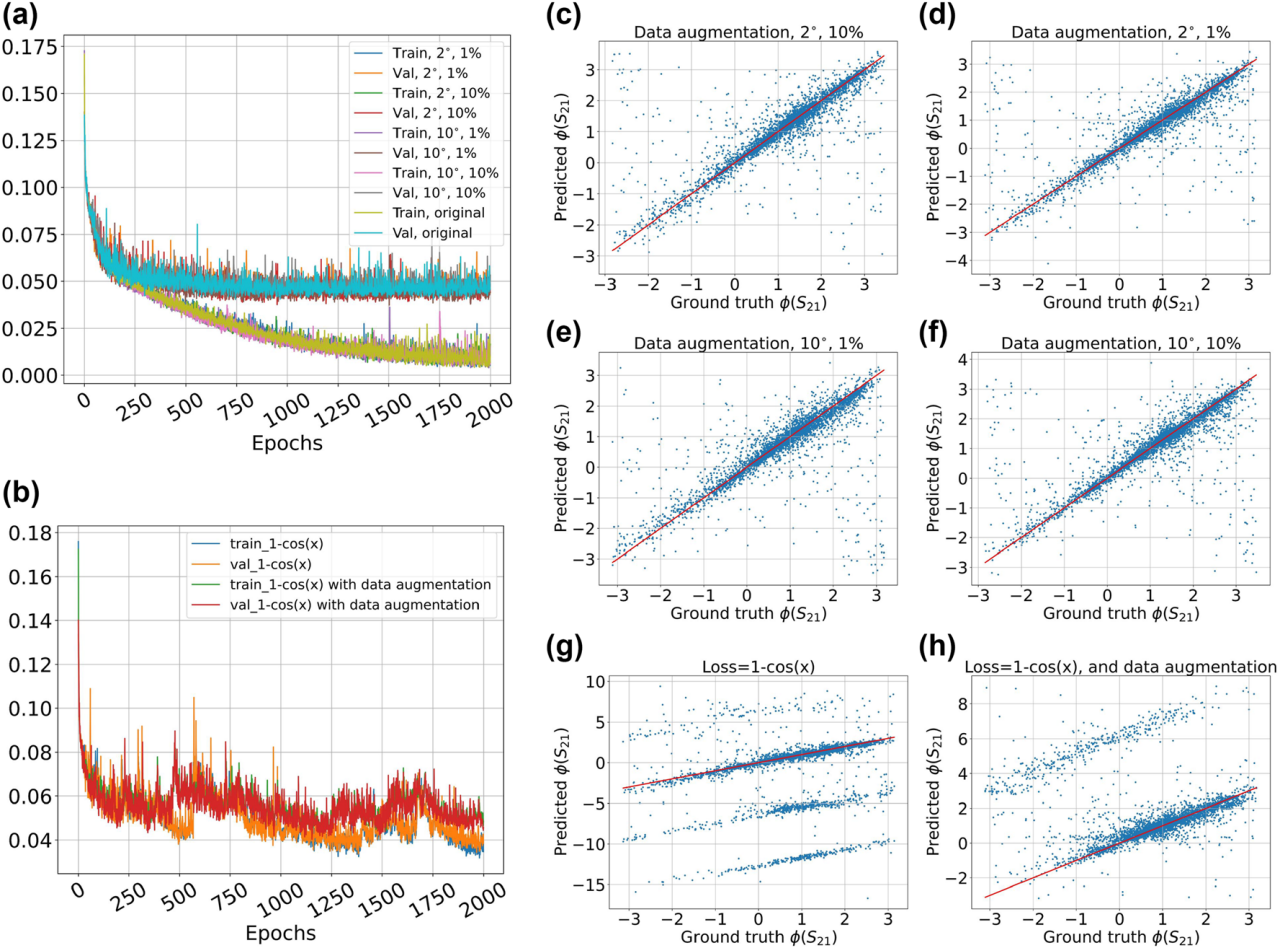
The subfigures demonstrate the performance of different methods to solve the scattering phase retrieval problem. (a) Demonstrates the train and validation loss curves of the data augmentation method to solve the problem of phase retrieval. In the figure the curves at the bottom indicate the training loss and the ones above are all showing validation loss. These curves do not align, pointing towards an overfitting problem, which is problematic for the inverse design purpose. (b) Shows the loss curves of another method in which we use a cosine similarity loss function (1 – cos $\left(\Phi_{S_{21}}^{\text{pred}}-\Phi_{S_{21}}^{\text{ground}}\right)$ ). Although, we do not face the problem overfitting problem here, the end points of the loss curves are not sufficiently low. (c)–(h) Demonstrate scatter plot of applying a test set to networks explained in (a) and (b). The majority of the points are gathered around the red bisector, however there is still a significant number of points, located too far away from this target.

In the data augmentation method, the phase values in the data set are shifted by small amounts according to the following relation:

(10) $$\Phi_{\text{shifted}}=\Phi_{S_{21}}+N\left(\pi ,\sigma^{2}\right)\times a,$$

where the noise to the phase $\Phi_{S_{21}}$ is a small value, multiplied with a sample from a Gaussian distribution $\left(N\left(\pi ,\sigma^{2}\right)\right)$ centered around *π* with few degrees standard deviation. The parameter “*a*” thus gives us freedom to determine how much we intend to alter the phase value in percentage. It is probable that the data augmentation improves the network generalization ability. Figure 4c–f demonstrate the scatter plots of the predicted phase of *S*_21_ over the ground truth values resulting from this data augmentation method. The values of the parameter “*a*” (in percentage) and standard deviation (in degrees) are different for each subfigure.

Another method that we used to tackle the phase retrieval problem is to exploit an alternative loss function to alleviate the phase discontinuity. We used the cosine similarity function with mathematical formulation:

(11) $$L=1-\cos\left(\Phi_{S_{21}}^{\text{pred}}-\Phi_{S_{21}}^{\text{ground}}\right).$$

The scatter plot associated with this method is shown in Figure 4g. The figure indicates that the DNN is not capable of properly representing the data. Finally, the result of combining the data augmentation and loss function alteration is also shown in Figure 4g. The plot shows small improvement, but generally it is not a very useful model for retrieving the phase. Table 5 includes the list of statistical measures for *S*_21_ phase models using the data augmentation and alternative loss function. It is evident from the table content that the augmentation method with *σ* = 2°, and *a* = 1 % possesses better statistical qualities, particularly compared to using cosine similarity loss function.

**Table 5:** The list of the statistical measures for the assessment of the DNN model for the prediction of Φ(*S*_21_) by utilizing data augmentation and alternative loss function. MAE, MSE, and EVS stand for mean-absolute error, mean-squared error, and explained-variance score, respectively.

	MAE	MSE	*R* ^2^ score	EVS
Triple-output DNN	0.1871	0.3107	0.7256	0.7256
Data augmentation *σ* = 2°, *a* = 1 %	0.215892	0.331471	0.707396	0.707733
Data augmentation *σ* = 2°, *a* = 10 %	0.222337	0.343684	0.696625	0.696626
Data augmentation *σ* = 10°, *a* = 10 %	0.230801	0.412481	0.635953	0.636698
Data augmentation *σ* = 10°, *a* = 1 %	0.257033	0.432282	0.618382	0.622001
Loss = 1 − cos $\left(\Phi_{S_{21}}^{\text{pred}}-\Phi_{S_{21}}^{\text{ground}}\right)$ , and data augmentation	0.448349	0.958009	0.154323	0.196733
Loss = 1 − cos $\left(\Phi_{S_{21}}^{\text{pred}}-\Phi_{S_{21}}^{\text{ground}}\right)$	0.650124	1.730549	−0.527622	−0.388227

**Figure 5:**
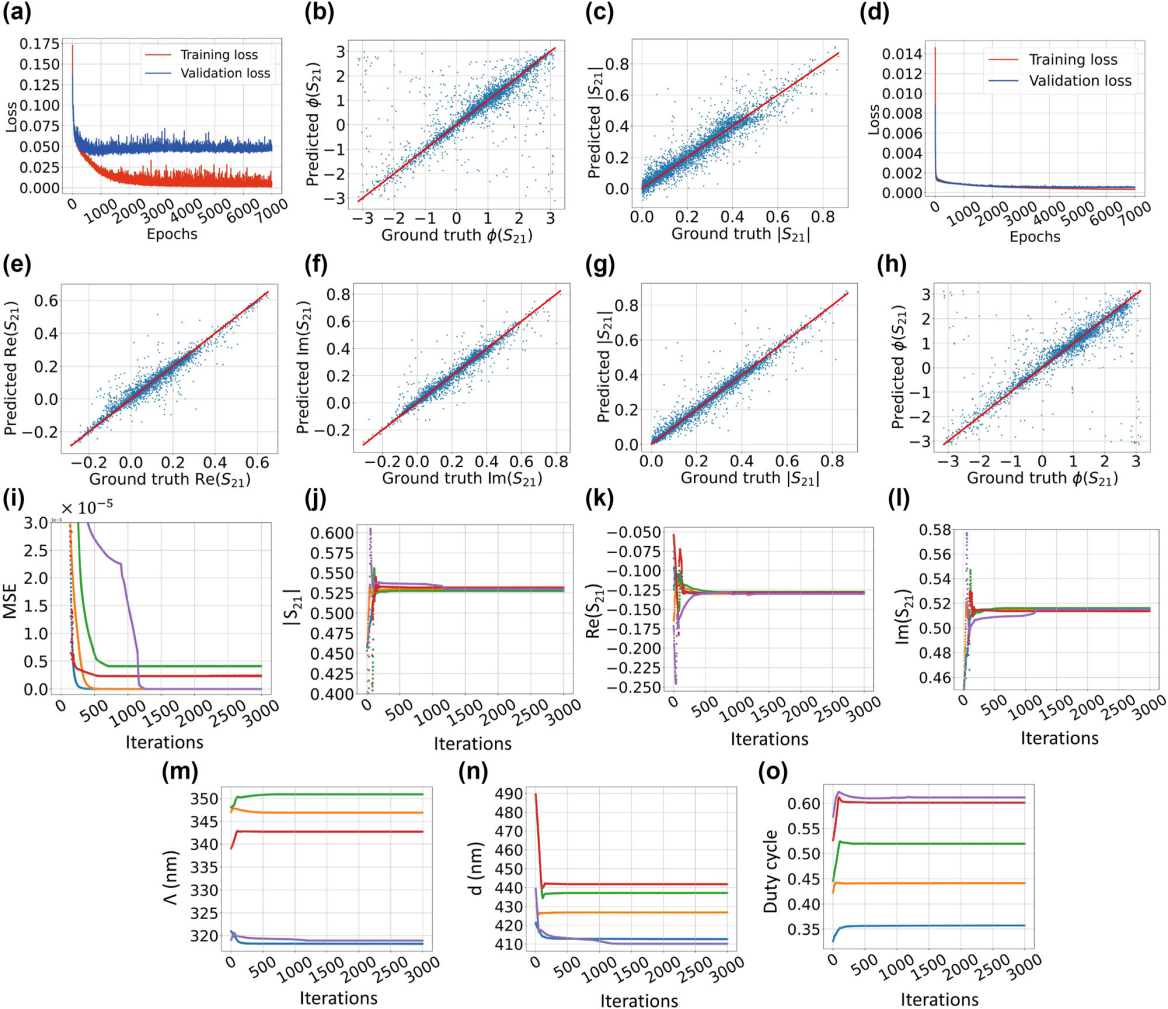
There are two groups of plots. The first group (a–h) shows the performance of a triple-output network designed to output the real part, imaginary part and the magnitude of one of the scattering parameters. The subfigures in the second group (i–o) illustrate the evolution of various parameters during the inverse design process for solving the phase retrieval problem. (a) Shows double-output DNN training and validation losses over epoch number. The neural network maps the mode converter physical properties to *S*_21_ amplitude and phase. (b) and (c) Show scatter plots predicted vs ground truth values of the *S*_21_ phase and amplitude for a test set applied to the neural network mentioned in (a). (d) Demonstrates the loss curves associated with the triple-output DNN. The output nodes are Re(*S*_21_), Im(*S*_21_), and |*S*_21_|. (e)–(g) Illustrate the scatter plots of the test set applied to triple-output neural network mentioned in (c). These subfigures are representing the Re(*S*_21_), Im(*S*_21_), and |*S*_21_|. (h) Shows the scatter plot of the phase Φ (*S*_21_) calculated from the scatter points in subfigures (e) and (f). The phase is calculated by taking inverse tangent of the real and imaginary parts of *S*_21_. (i)–(o) Demonstrate results obtained from the inverse design of the grating mode converter for desired values Re(*S*_21_) = −0.13, Im(*S*_21_) = 0.515, and |*S*_21_| = 0.53. The inverse design algorithm is used several times leading to five different solutions. Each colored curve in the figures is assigned to the same inverse design solution.

A different method for DNN modeling of the phase includes creating a neural network model that links the input features to the real part, imaginary part, and magnitude of the scattering parameter. In this scenario, the neural network model must possess a high level of accuracy in estimating both the real and imaginary components. This aids in reducing the error in the ratio between the imaginary and real components. In this case, the DNN has the same configuration as the ones mentioned in the previous sections. Figure 5d demonstrates the training and validation losses over epoch number for the DNN model with output nodes Re(*S*_21_), Im(*S*_21_), and |*S*_21_|. The curves clearly exhibits low values for the losses in the final epochs. In Table 6, the statistical measures calculated for triple-output DNN are listed. All numbers are adequately qualified to claim that the DNN exhibits proper performance. Figure 5e–g demonstrate the scatter plots of the predicted over the ground truth values of the test set for Re(*S*_21_), Im(*S*_21_), and |*S*_21_|, respectively.

**Table 6:** The list of the statistical measures for the assessment of the triple-output DNN model for the prediction of Re(*S*_21_), Im(*S*_21_), and |*S*_21_|. MAE, MSE, and EVS stand for mean-absolute error, mean-squared error, and explained-variance score, respectively.

	MAE	MSE	*R* ^2^ score	EVS
Re(*S*_21_)	0.0155	0.0009	0.9468	0.9468
Im(*S*_21_)	0.0156	0.0011	0.9559	0.9559
|*S*_21_|	0.0144	0.0009	0.9663	0.966370

Exploiting the values shown in Figure 5e and f, we are able to calculate the scatter plot points for the *S*_21_ phase, which is shown in Figure 5h. Compared to the case illustrated in Figure 5b (associated with the double-output network), the Figure 5h exhibits improvement, since more scattered points are closer the middle ideal line. In terms of the statistical measures, the EVS, *R* ^2^ score, and MSE values for this case are, respectively, equal to 0.72, 0.72, and 0.31. This shows some improvement compared to case of double-output DNN. Furthermore, the triple-output DNN also improves the statistical measures associated with |*S*_21_| (Table 6) in comparison with the double-output DNN (EVS = 0.913, *R* ^2^ score = 0.913, MSE = 0.0024). Table 5 contains all statistical information to compare the results from the triple-output DNN with the data augmentation and using alternative loss function. It is evident that triple-output exhibits better results. This observation is also found by comparing Figures 5h with 4c–h, where the scattered points are more distant from the ideal red line.

There are other advanced methods capable of modeling the complex behavior of such phase data. Physics-informed neural networks (PINNs) is a method that the Maxwell’s equations get involved into the training process providing the physical constraints on the problem that ultimately assist in generalization capability of the network [46]. A hybrid utilization of adjoint methods and deep neural networks is also a powerful method, where the network performs rapid approximation, and the adjoint solver performs fun-tuning the phase accuracy [47]. Now, based on the trained triple-output DNN, it is possible to perform inverse design of the mode converter to achieve pre-defined Re(*S*_21_), Im(*S*_21_), and |*S*_21_|. The method is the same as the one discussed in Sections 3 and 4. The primary advantage of this inverse design is the ability to choose the real and imaginary components of the associated scattering parameter depending on the desired phase value of the scattering parameter. Hence, the results of the inverse design process consist of the specific geometric characteristics of the grating that not only generate the intended real and imaginary components of the scattering parameter, but also achieve the correct phase.

For the present case, the selected desired values are Re(*S*_21_) = –0.13, Im(*S*_21_) = 0.515, and |*S*_21_| = 0.53. Based on the values of the real and imaginary parts, the desired phase of *S*_21_ is equal to *ϕ*(*S*_21_) = 104.17°. Figure 5e–j demonstrate the results of the inverse design. Table 7 lists the numerical results of the inverse design. It is evident from the last column of the Table that the results are very close to the desired phase value *ϕ*(*S*_21_) = 104.17°.

**Table 7:** The following table presents the values of the physical characteristics of the grating (first 3 columns) determined using the inverse design process for the desired scattering values Re(*S*_21_) = −0.13, Im(*S*_21_) = 0.515, and |*S*_21_| = 0.53. The computed scattering parameters through the COMSOL software are indicated by the columns denoted by the subscript “*c*”. The columns denoted by subscript “*m*” are the estimated results from the triple-output DNN model. The column denoted by *ϕ*(*S*_21*c*_) indicates the phase calculated by using the values in Re(*S*_21*c*_) and Im(*S*_21*c*_) columns. Moreover, the column indicated by *ϕ*(*S*_21*m*_) is the phase calculated from the values Re(*S*_21*m*_) and Im(*S*_21*m*_) columns. The desired value of the phase based on Re(*S*_21_) = −0.13 and Im(*S*_21_) = 0.515 is equal to 104.17°. The colored rows are relevant to the inverse design solution curves in Figure 5i–o.

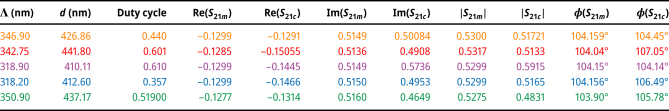

## 6 Conclusions

In this article, we exploit the power of deep neural networks to perform inverse design of the waveguide grating mode converter. For the inverse design goal, we first establish various forward models based on deep neural networks for the grating mode converter. The required data to train the neural networks is obtained through the simulation of the grating structure by the finite element software COMSOL Multiphysics. The input nodes of the neural networks are the grating period, corrugation depth, and the duty cycle. The neural networks outputs are considered to be magnitudes and the phases of the scattering parameters. The scattering parameters are the reflection coefficients of the converted modes reflected from the grating.

There are two layers of complexity to consider when scaling the proposed technique: As the number of supported optical modes increases, the number of possible mode conversions grows combinatorially, as discussed in previous work [32]. The second level of complexity involves the design and optimization of the individual mode converters and their integration within a deep neural network-based inverse design framework. This was the focus of our current study. The computational complexity of our inverse design methodology scales with the number of trainable parameters in the neural network. As the number of guided optical modes increases, the dimensionality of the scattering parameter space also grows, requiring a more complex neural network architecture with a larger dataset for training. However, our approach benefits from the ability of deep learning models to generalize efficiently across large design spaces. Our trained models have demonstrated high accuracy even for multi-output regression tasks, as shown in the case of triple-output networks predicting multiple scattering parameters. The computational cost of each inverse design process is largely independent of the number of modes. However, generating sufficient training data through electromagnetic simulations (e.g., using COMSOL) will become more time-intensive as more modes are considered. This can be computationally expensive, especially for complex photonic structures where each data point requires solving Maxwell’s equations with fine spatial resolution. While transfer learning and data augmentation can partially mitigate this issue, the initial cost of dataset generation remains a constraint. Computational cost is another factor, as deep neural networks, particularly those with large architectures, require significant GPU or cloud computing resources for training. However, once trained, the network provides near-instantaneous predictions, making it highly advantageous over traditional iterative optimization methods for large-scale inverse design problems.

Finally, there’s also a subtlety related to the different design parameters. In particular the waveguide thickness is a crucial design parameter, because its variations influence the system in two fundamental ways. First, thickness fluctuations alter the effective refractive index of the guided modes, modifying their propagation constants. Since mode conversion relies on phase matching, even small deviations in thickness can cause phase mismatches and reduce conversion efficiency. In extreme cases, thickness variations may even cause certain modes to disappear, fundamentally changing the mode structure of the system. Second, the scattering properties of the grating-based mode converter depend sensitively on the waveguide thickness. A small shift in thickness alters the reflection/transmission coefficients and changes the relative amplitudes and phases of the converted modes. This can degrade the precision of the designed grating, leading to unexpected behavior in multi-mode interference and cascaded mode conversion systems. Thus, controlling and accounting for thickness variations is essential for ensuring robust inverse design. All libraries and codes developed and used in this manuscript are available in a link in Supplementary Materials. With these efforts, we want to provide the photonics community with a tool for controlling the phases the magnitudes of the multiple waves reflected from integrated mode converters. This capability could be utilised in interference of multiple counter-propagating waves or advanced integrated photonic circuits.
